# Identification of winter moth (*Operophtera brumata*) refugia in North Africa and the Italian Peninsula during the last glacial maximum

**DOI:** 10.1002/ece3.5830

**Published:** 2019-11-18

**Authors:** Jeremy C. Andersen, Nathan P. Havill, Yaussra Mannai, Olfa Ezzine, Samir Dhahri, Mohamed Lahbib Ben Jamâa, Adalgisa Caccone, Joseph S. Elkinton

**Affiliations:** ^1^ Department of Environmental Conservation University of Massachusetts Amherst Amherst MA USA; ^2^ Northern Research Station USDA Forest Service Hamden CT USA; ^3^ LR161INRGREF01 Laboratory of Management and Valorization of Forest Resources National Institute for Research in Rural Engineering Water and Forest (INRGREF) University of Carthage Ariana Tunisia; ^4^ LR161INRGREF03 Laboratory of Forest Ecology National Institute for Research in Rural Engineering Water and Forest (INRGREF) University of Carthage Ariana Tunisia; ^5^ Department of Ecology & Evolutionary Biology Yale University New Haven CT USA

**Keywords:** approximate Bayesian computation, geometridae, microsatellites, North African region, phylogeography, population genetics, postglacial recolonization

## Abstract

Numerous studies have shown that the genetic diversity of species inhabiting temperate regions has been shaped by changes in their distributions during the Quaternary climatic oscillations. For some species, the genetic distinctness of isolated populations is maintained during secondary contact, while for others, admixture is frequently observed. For the winter moth (*Operophtera brumata*), an important defoliator of oak forests across Europe and northern Africa, we previously determined that contemporary populations correspond to genetic diversity obtained during the last glacial maximum (LGM) through the use of refugia in the Iberian and Aegean peninsulas, and to a lesser extent the Caucasus region. Missing from this sampling were populations from the Italian peninsula and from North Africa, both regions known to have played important roles as glacial refugia for other species. Therefore, we genotyped field‐collected winter moth individuals from southern Italy and northwestern Tunisia—the latter a region where severe oak forest defoliation by winter moth has recently been reported—using polymorphic microsatellite. We reconstructed the genetic relationships of these populations in comparison to moths previously sampled from the Iberian and Aegean peninsulas, the Caucasus region, and western Europe using genetic distance, Bayesian clustering, and approximate Bayesian computation (ABC) methods. Our results indicate that both the southern Italian and the Tunisian populations are genetically distinct from other sampled populations, and likely originated in their respective refugium during the LGM after diverging from a population that eventually settled in the Iberian refugium. These suggest that winter moth populations persisted in at least five Mediterranean LGM refugia. Finally, we comment that outbreaks by winter moth in northwestern Tunisia are not the result of a recent introduction of a nonnative species, but rather are most likely due to land use or environmental changes.

## Graphical abstract

Here, we find the use of last glacial maximum refugia in southern Italy and North Africa by the forest defoliator winter moth. This suggests that winter moth populations persisted in at least five Mediterranean LGM refugia. Finally, we comment that outbreaks by winter moth in northwestern Tunisia are not the result of a recent introduction of a nonnative species, but rather are most likely due to land use or environmental changes.

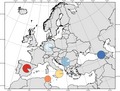

## 1 INTRODUCTION

Natural movements of organisms following the expansions and contractions of glacial ice sheets during the Quaternary climatic oscillations have resulted in the geographic and genetic isolation of species/populations across temperate regions (Hewitt, 1996, 2000; Schmitt, Rober, & Seitz, 2005; Schmitt & Seitz, 2001). For example, in Europe, many studies have shown how the persistence of populations in separate glacial refugia (e.g., the Iberian, Italian, and Balkan peninsulas, the Caucasus region, North Africa, and microrefugia in the Alps) during the Quaternary climatic oscillations promoted genetic diversity in contemporary populations (Andersen, Havill, Caccone, & Elkinton, 2017; Habel, Lens, Rodder, & Schmitt, 2011; Huck, Buedel, & Schmitt, 2012; Niedzialkowska et al., 2016; von Reumont, Struwe, Schwarzer, & Misof, 2012; Rofes et al., 2018; Schmitt, 2007; Schmitt & Müller, 2007; Sim, Hall, Jex, Hegel, & Coltman, 2016; Stolting et al., 2015; Torroni et al., 2001). For outbreaking species, reconstructing the biogeographical history of the species can help to determine the factors that might be promoting their outbreak (e.g., Moyal et al., 2011; Song et al., 2018; Zhu et al., 2016).

One species that frequently reaches outbreak densities across much of its native range of Europe and western Asia (Elkinton, Boettener, Liebhold, & Gwiazdowski, 2015) is the winter moth, *Operophtera brumata* (Lepidoptera: Geometridae). Throughout its distribution, winter moth is a serious pest of many broadleaf tree and shrub species, including oak (*Quercus* spp.), maple (*Acer* spp.), and birch (*Betula* spp.) trees (Wint, 1983). Winter moth is also present in North Africa, where it was first reported in the 1970s (Ferguson, 1978; Hausmann & Viidalepp, 2012). More recently it has been observed at outbreak densities in oak forests (*Quercus* spp.) in northwestern Tunisia beginning in 2009 (Mannai, Ezzine, Nouira, & Ben Jamâa, 2015), though the reasons for these outbreaks are currently unknown. In this region, mature oak forests extend mainly to the regions of Kroumirie and Mogods, from the Algerian–Tunisian border in the west, to Lake Ichkeul in the east (Debazac, 1959; DGF, 2005), and are home to five oak species: *Quercus suber* L., *Quercus canariensis* Willd, *Quercus ilex* L., *Quercus coccifera* L., as well as a Tunisian–Algerian endemic species *Quercus afares* Pomel (Boudy, 1950). In Tunisia, winter moth has mainly been observed on *Q. canariensis* in both mixed oak (Aïn Zena, 36.435°N, 8.515°E) and pure *Q. canariensis* oak (Mzara Forest, 36.769°N, 8.72°E) forests, with lower densities being found on *Q. afares* and *Q. suber* compared with *Q. canariensis* in Aïn Zena. These differences may be the result of differences in budburst timing of different oak species (Mannai, Ezzine, Hausmann, Nouira, & Ben Jamâa, 2017).

As a result of the movement of nursery stocks, invasive populations of winter moth have also been introduced to North America (Ferguson, 1978), with populations established in Nova Scotia in the 1920s (MacPhee, 1967), Oregon in the 1950s (Kimberling, Miller, & Penrose, 1986), British Columbia in the 1970s (Gillespie, Finlayson, Tonks, & Ross, 1978), and in the northeastern United States in the 1990s (Elkinton et al., 2015). Therefore, we were curious as to whether or not the recent reports of outbreaks in northwestern Tunisian oak forests were the result of a recent introduction (as has been the case in North America), or whether a native population has achieved outbreak levels for some other reason. Previously, Mannai et al. (2017) explored the native/nonnative status of outbreaking Tunisian winter moth populations by sequencing a fragment of the mitochondrial (mtDNA) locus cytochrome oxidase I (COI) from field‐collected larvae in Tunisian oak forests, and compared these sequences to those published in NCBI GenBank and the Barcode of Life Data Systems (BOLD) database. This work confirmed the field identifications of winter moth in northwestern Tunisia. Unfortunately, due to the overall lack of genetic diversity of this COI fragment, the native/nonnative status of these populations could not be determined. This low diversity in COI was similarly observed by Gwiazdowski, Elkinton, deWaard, and Sremac (2013) where winter moth samples collected from across Europe were predominantly assigned to two haplotypes that differed by only two substitutions (~0.5% divergence), thereby hampering the ability of COI data alone to determine relationships among populations.

Previously, we examined the use of glacial refugia during the last glacial maximum (LGM) by winter moth (Andersen et al., 2017). In that work, we genotyped field‐collected individuals from across Europe with 24 highly polymorhic microsatellie loci. In contrast to mtDNA sequence data that showed limited biogeographic structure between winter moth populations, we found evidence that individuals could be assigned to either an eastern or a western European genetic lineage, with a clear hybrid zone in central Europe (Andersen et al., 2017). Using approximate Bayesian computation (ABC) methods, we also hypothesized that the contemporary genetic diversity of European populations of this species was largely shaped by its retreat to refugia in the Iberian, Balkan, and Caucasus regions, and by hybridization between the Iberian and Balkan lineages postrecolonization.

Absent from the sampling in Andersen et al. (2017), however, were moths from the Italian peninsula and from North Africa, both regions known to have acted as glacial refugia for other species (see Hewitt, 2000; Schmitt, 2007). Therefore, in this study we obtain new winter moth samples from Tunisia, Italy, and additional specimens from Spain, to further explore the biogeographic history of winter moth and its use of potential glacial refugia during the LGM. Specifically, our research objectives were as follows: (a) to genotype winter moth individuals collected in potential glacial refugia not sampled in Andersen et al. (2017; i.e., Southern Italy and North Africa), and (b) to determine whether outbreaking populations of winter moth in Tunisia represent a native population dating back to the LGM or whether they were recently introduced to the region, and if so from where.

## 2 METHODS

### 2.1 Data collection

Adult male winter moths were collected using pheromone‐baited traps (Elkinton et al., 2010; Elkinton, Lance, Boettner, Khrimian, & Leva, 2011) deployed in Barcelona, Catalonia, Spain, and in Ortì, Calabria, Italy. After moths were collected from the traps, they were placed in glassine envelopes (Uline Corporation, USA), and stored at −80°C. In addition, we sampled larval winter moth collected from trees in Mzara Forest, Tunisia (pure forest of *Q. canariensis*, alt 653 m.). Once at the laboratory, larvae were placed in 96% ethanol, and then stored at −80°C prior to molecular analyses. See Table 1 for complete locality information. To this dataset, we added genotype scores from individuals presented in Andersen et al. (2017), including all 28 individuals from Tbilisi, Republic of Georgia, 30 randomly selected individuals from Germany, 30 randomly selected individuals from Serbia, and 20 randomly selected individuals from Spain (bringing the total for Spanish samples to 30).

**Table 1:** Locality collection information including accession numbers, the number of samples collected (*N*), the collector, collection date (Date), and geographical coordinates (i.e., latitude and longitude) in decimal–degree format

Country	Location	*N*	Collector	Date	Latitude	Longitude
Georgia	Tbilisi	28	G. Japoshvili	2xii2015	41.8068	44.7268
Germany	Bühren	6	F. Krueger	18xii2006	51.4658	9.6778
Germany	Göttingen	8	F. Krueger	7i2007	51.5460	9.9111
Germany	Reinhardshagen	8	F. Krueger	22xi2006	51.4763	9.5154
Germany	Schlüchtern	8	F. Krueger	20xii2006	50.2425	9.4538
Italy	Ortì	26	E. Castiglione and F. Manti	11ii2018	38.1475	15.7196
Serbia	Belgrade	9	M. Glavendekić	27xi2006	44.7643	20.4364
Serbia	Pančevo	11	M. Glavendekić	4xii2006	44.8524	20.6532
Serbia	Pančevo	10	M. Glavendekić	28xi2006	44.8524	20.6532
Spain	Barcelona	10	J. Bau	12i2018	41.9657	2.1907
Spain	La Langa	2	A. Pepi	6xii2015	40.0895	−2.6607
Spain	Uriz	1	M. Lombardero	xii2008	43.1184	−7.6517
Spain	Uriz	1	M. Lombardero	xii2008	43.1184	−7.6517
Spain	Lugo	1	M. Lombardero	xii2008	42.9922	−7.5451
Spain	Uriz	1	M. Lombardero	xii2008	43.1190	−7.6497
Spain	Uriz	1	M. Lombardero	xii2008	43.1190	−7.6497
Spain	Uriz	3	M. Lombardero	xii2008	43.1184	−7.6517
Spain	Uriz	1	M. Lombardero	xii2008	43.1190	−7.6497
Spain	Lugo	1	M. Lombardero	xii2008	42.9922	−7.5451
Spain	Lugo	8	M. Lombardero	9i2014	42.9926	−7.5441
Tunisia	Mzara Forest	17	O. Ezzine	27iv2018	36.7690	8.7200

### 2.2 DNA extraction and microsatellite amplification

Whole genomic DNA was extracted using the Omega Bio‐tek E.Z.N.A.^®^ Tissue DNA Kit (Omega Bio‐tek) following the manufacturer's instructions. For adult males, prior to DNA extraction, the uncus and wings were removed and stored as morphological vouchers. Vouchers were deposited at the Yale Peabody Museum of Natural History, New Haven, Connecticut, USA. Specimen was then homogenized using 3/16″ stainless steel beads (GlenMills Inc.) using a FastPrep‐24 Sample Homogenizer (MP Biomedicals). Twenty‐four polymorphic microsatellite loci were amplified following protocols presented in Havill et al. (2017), and genotyping was performed at the DNA Analysis Facility on Science Hill at Yale University in comparison to the GeneScan 500 LIZ size standard (Thermo Fisher Scientific) using a 3730xl DNA Analyzer (Thermo Fisher Scientific). Fragment lengths were scored using the microsatellite plugin in the software program geneious v. R11 (https://www.geneious.com). To address potential variation in allele fragment lengths generated during different genotyping runs (see Hoffman & Amos, 2005), the same laboratory methods, personnel, sequencing instrument, and allele bins in geneious were used for all runs, including those performed by Andersen et al. (2017). Individuals for which ≥20 microsatellite loci were successfully amplified were included in subsequent analyses. This subset included up to 30 randomly selected individuals from each of the following locations (a) Spain, (b) Serbia, (c) Republic of Georgia, and (d) Germany. Locality collection information for each individual in the analyses is provided in Table 1, and microsatellite genotype information is as a tab‐delimited supplemental file titled “WinterMothTunisiaStructure.txt.”

### 2.3 Bayesian genetic clustering

The assignment probabilities (*Q*) of each individual to distinct genetic clusters (*K*) were estimated using the Bayesian genetic clustering software structure v.2.3.2 (Falush, Stephens, & Pritchard, 2003; Pritchard, Stephens, & Donnelly, 2000) for values ranging from *K* = 1 to *K* = 8. For each value of *K,* ten independent analysis were run, each with random starting values, a burn‐in period of 100,000 generations, and a total runtime of 1,000,000 generations. For each run, we utilized the admixture model, with correlated allele frequencies using default settings for both. The optimal number of clusters was then estimated using the Delta‐K method of Evanno, Regnaut, and Goudet (2005) as implemented in the software package structureharvester (Earl & vonHoldt, 2012), and values for *Q* were averaged across runs using Clumpak (Kopelman, Mayzel, Jakobsson, Rosenberg, & Mayrose, 2015).

### 2.4 Genetic diversity and differentiation

For individuals from each country, we calculated several population genetic statistics, including the number of individuals genotyped (*n*), the average number of alleles per locus (*Na*), the effective number of alleles (*Eff_Na*), the average observed heterozygosity across loci (*H*_o_), the average heterozygosity within each population (*H*_s_), the total heterozygosity (*H*_t_), the average inbreeding coefficient (*G*_IS_), and the presence of deviation from Hardy–Weinberg Equilibrium using genodive v.2.0b27 (Meirmans & van Tienderen, 2004). The proportion of null alleles present in the dataset and “excluding null alleles” (ENA)‐corrected measures of genetic differentiation (*F*_ST_) among populations from each country was estimated were estimated using FreeNA (Chapuis & Estoup, 2007), the subsequent pairwise corrected *F*_ST_ distance matrix was then used to calculate a “NeighborNet” network with the program SplitsTree v.4.14.2 (Huson & Bryant, 2006). The probability of population differentiation was then estimated using GenePop (Raymond & Rousset, 1995; Rousset, 2008).

### 2.5 Historical demography

To estimate the divergence time between the Tunisian population and Eurasian populations of winter moth using ABC as implemented in the software DiyABC v.2.1.0 (Cornuet et al., 2014, 2008), we used a two‐step approach to limit the number of potential scenarios (see Stone et al., 2017 for an example). In the first step, we first estimated the relationship of the southern Italian population to other European populations (i.e., Georgian, German, Serbian, and Spanish). We used the results from Andersen et al. (2017) to establish the relationships among the other European populations. Andersen et al. (2017) found strong support for the German lineage to be the result of admixture between the Spanish and Serbian lineages after the LGM. The authors were unable, however, to conclude whether the Serbian and Georgian lineages diverged before or after the divergence of the Spanish lineage, as these two scenarios received equivocal results. To reduce complexity in scenario design, here we use a tree with the Spanish lineage diverging prior to the divergence of the Serbian and Georgian lineages [Scenario 7 from Andersen et al. (2017)] as our root tree. Three scenarios (Figure S1) were compared to determine whether the Italian population was most closely related to the Spanish, Serbian, or Georgian populations by generating a reference table based on three million simulated datasets (one million per scenario).

The branching pattern among European populations from the scenario receiving the highest support from the first step was then used to explore the origins of the Tunisian population. Nine scenarios were explored (Figure S2); four scenarios for the Tunisian population diverging from the Spanish, southern Italian, Serbian, and Georgian populations with branching times corresponding with the LGM, and five scenarios for the Tunisian population diverging from the Spanish, southern Italian, Serbian, and Georgian as well as from the admixed German population with a branching time corresponding to contemporary establishment (i.e., within the last ~1,000 years). The contemporary scenarios also included an additional population size parameter to allow for changes in population size following the divergence from potential source populations (i.e., to allow the possibility of a genetic bottleneck). Initially, three additional scenarios were explored (not shown) where the Tunisian population acted as a source for the Spanish and Italian populations; however, these scenarios received posterior probabilities of 0.0000 in comparison to a European origin of the Tunisian population (poster probability of 1.0000), and such “out‐of‐Africa” scenarios were not pursued further.

All scenarios for both steps included the following: (a) multiple population size parameters to allow for changes in population sizes following splitting/merging events; (b) default mutation model parameters, except that the minimum mean mutation rate to 1 × 10^−5^, and maximum values for the mean and individual locus coefficient Ps were both set to 1.0; and (c) four loci with especially large allelic ranges (2,339, 925, 2,191, and 12,042) were removed to improve the shape of the cloud of simulated datasets. For both sets of simulations, the following summary statistics were used: one sample; mean number of alleles, mean genic diversity, mean size variance: two sample; *F*_ST_, classification index, and (dμ)^2^ distance. Distributions for parameter priors are presented in Table 3. The scenarios were then compared using and the “Logistic Regression” tests as part of the “Compute posterior probabilities of scenarios” analysis in DiyABC based on comparisons of 1% of simulated datasets closest to the observed data (Cornuet, Ravigné, & Estoup, 2010; Cornuet et al., 2008). To determine the goodness of fit of simulated scenarios, we first conducted a principle components analysis (PCA) that examined the posterior distributions of summary statistics in comparison to the observed dataset, as implemented in the “Perform model checking” analysis in DiyABC. We then estimated error rates by implementing the “Evaluate confidence in scenario choice” analysis in DiyABC.

## 3 RESULTS

### 3.1 Data collection, DNA extraction, and microsatellite amplification

After filtering individuals from which less than 20 of 24 polymorphic microsatellites were amplified, ten winter moth individuals collected in Barcelona, Spain, 26 individuals from Ortì, Italy, and 17 individuals from Mzara Forest, Tunisia were included in subsequent analyses.

### 3.2 Bayesian genetic clustering

**Figure 1:**
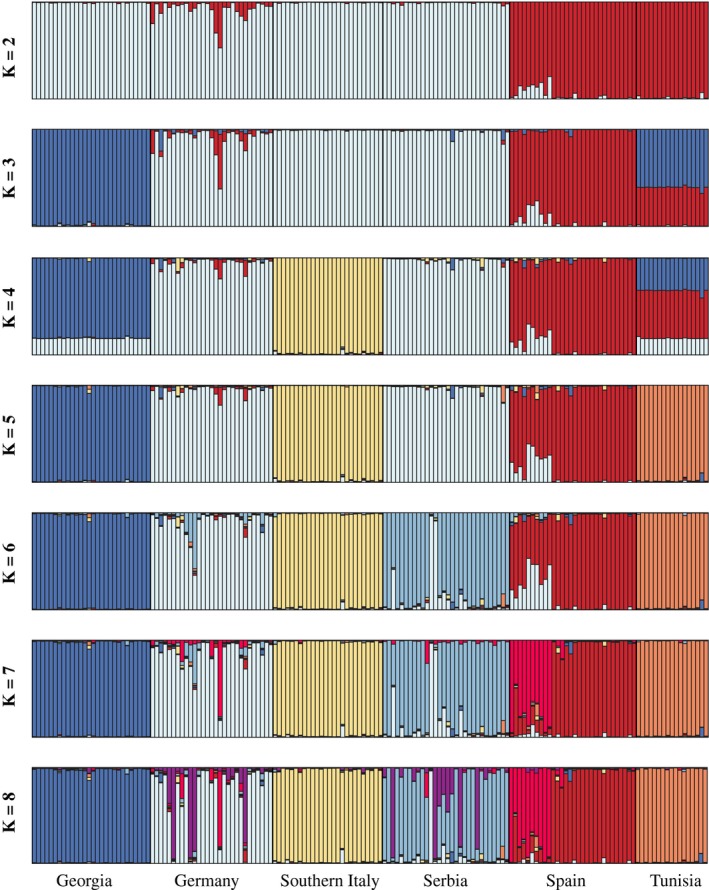
Proportional assignment (*Q*) of individuals to genetic clusters (*K*) based on summary across independent Structure runs using Clumpak

**Figure 2:**
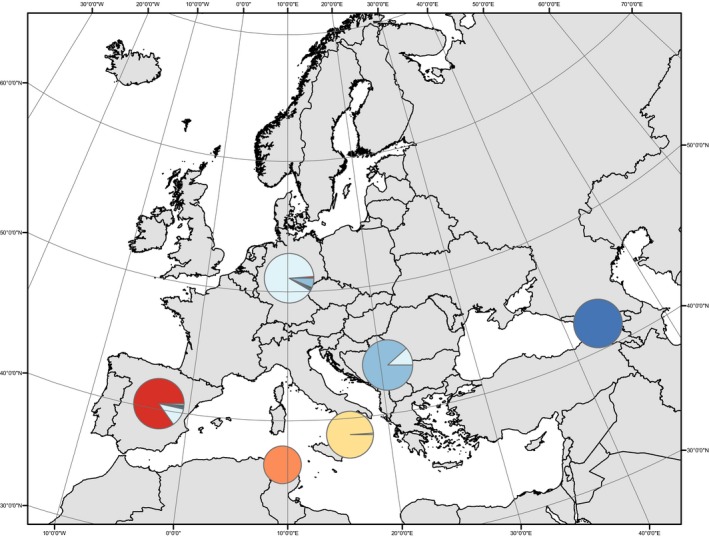
Population genetic structure of winter moth. Assignment probabilities (*Q*) of individuals for *K* = 6 as average by country of collection. Pie‐charts are sized proportional to the number of sampled individuals in each population and are centered on the country of collection, except for the Ortì, Italy, and Mzara Forest, Tunisia populations which are centered on their sample localities. Results are displayed using the Europe Albers Equal Area Conic projection as implemented in arcmap v.10.3.1 (ESRI, Inc.)

The probability of assignment (*Q*) of individuals to major genetic clusters (*K*) for *K* = 1 through *K* = 8 after averaging across independent Structure runs is presented in Figure 1. Assignments for major and minor clusters, as well as the number of runs supporting each cluster, after summary in Clumpak are presented in Figure S3. Results from structureharvester indicated that the optimal number of genetic clusters (*K*) present in the dataset was *K* = 6 (Figure S4). At *K* = 6, all ten independent Structure runs were assigned to one major cluster using Clumpak, and these genetic clusters predominantly corresponded to the geographical origin of samples (Figures 1 and 2).

### 3.3 Genetic diversity and differentiation

**Figure 3:**
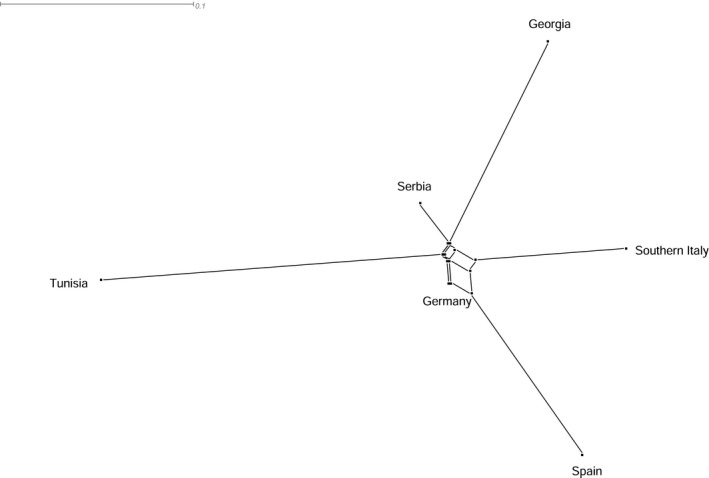
Schematic representation of *F*_ST_ distances between countries visualized as a NeighborNet in SplitsTree

Population genetic statistics for each population are presented in Table 2. The German population displayed the highest number of effective alleles (*Eff_Na* = 4.987) as well as the highest level of observed heterozygosity (*H*_o_ = 0.670) while the Mzara Forest, Tunisia population had the lowest number of effective alleles (*Eff_Na* = 2.817) and the lowest level of observed heterozygosity (*H*_o_ = 0.450). As per Andersen et al. (2017), all sampled localities displayed highly significant deviations from Hardy–Weinberg Equilibrium (*p* = .001 for all). The average value ± standard error (*SE*) for the null allele frequency was 0.053 ± 0.024. The average ENA‐corrected measure of *F*_ST_ pairwise differences among population pairs was 0.185 ± 0.02. Values for all pairwise comparisons are presented in Table S1. The NeighborNet analyses indicated that all sampled populations showed a high degree of genetic distinction, with all but the German population being placed at the end of a long branch (Figure 3).

**Table 2:** Population genetic diversity including the number of individuals genotyped (*n*), the average number of alleles per locus (*Na*), the effective number of alleles (*Eff_Na*), the average observed heterozygosity across loci (*H*_o_), the average expected heterozygosity within each population (*H*_s_), the total expected heterozygosity (*H*_t_), the average inbreeding coefficient (*G*_IS_), the presence of deviation from Hardy–Weinberg Equilibrium (HWE) using genodive, and the average null allele frequency (Null) estimated using FreeNA

Population	*n*	*Na*	*Eff_Na*	*H*_o_	*H*_s_	*H*_t_	*G*_IS_	HWE	Null
Tbilisi, Georgia	28	7.708	3.551	0.519	0.590	0.590	0.120	0.001	0.049
Germany	30	10.875	4.987	0.670	0.757	0.757	0.115	0.001	0.046
Ortì, Italy	26	8.250	4.341	0.592	0.670	0.670	0.117	0.001	0.047
Serbia	30	10.542	5.634	0.565	0.723	0.723	0.218	0.001	0.083
Spain	30	6.792	3.079	0.486	0.584	0.584	0.168	0.001	0.059
Mzara Forest, Tunisia	17	4.750	2.817	0.450	0.488	0.488	0.078	0.001	0.034

### 3.4 Historical demography

**Figure 4:**
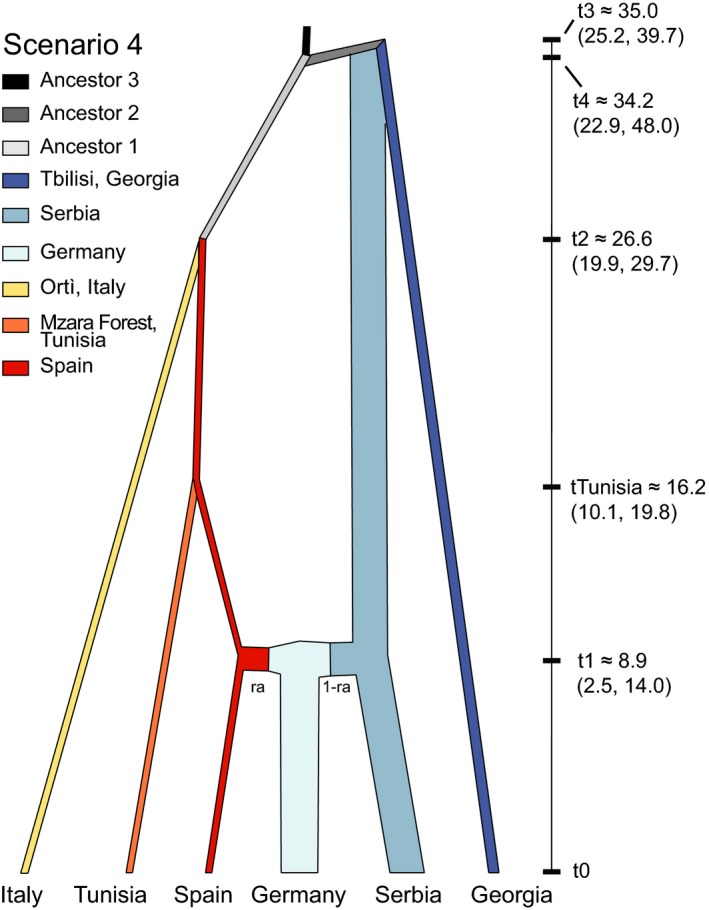
Evolutionary scenario of European and North African populations of winter moth based on simulations conducted in DiyABC. Lines widths are drawn relatively proportional to the mean estimated population size. At each node, the mean divergence/merger time is shown along the y‐axis (1 ka = 1,000 years). Ninety‐five percent confidence intervals for population sizes, divergence/merger times, and for the amount of admixture (ra) are presented in Table 3

Results from simulations conducted during the first step in our diyabc analyses indicated that the Ortì, Italy population was most closely related to the Spanish population (Figure S5 and Table S2). Model‐checking analyses for the posterior distributions for summary statistics based on this dataset formed a distinct cloud around the observed dataset (Figure S6). Evaluations of the confidence in the scenario choice indicated a posterior predictive error rate of 0.064. Results for simulations conducted as part of our second step of the analyses also indicated that the Tunisian population was most closely related to the Spanish one (Figure 4), and this result was strongly supported by the logistic regression test (Figure S7 and Table S3). Model‐checking analyses for the posterior distributions for summary statistics based on this dataset formed a distinct cloud around the observed dataset (Figure S8). Evaluations of the confidence in the scenario choice indicated a posterior predictive error rate of 0.287. Based on this scenario (Figure 4), the Tunisian population likely diverged from the Spanish population ~16,200 years ago (95% CI = 10,100–19,800 years ago; Table 3). All estimates for demographic parameters for the most likely scenarios were calculated based on the analysis of 1,000,000 simulated datasets and are presented in Figures S9 and S10, for the scenarios estimating the relationship of the southern Italian population and the Tunisian populations, respectively, and summarized for the Tunisian scenario in Table 4.

**Table 3:** Demographic parameter priors for DiyABC simulations

Parameters	Prior	
	Min	Max
Population sizes (effective population size)		
Tbilisi, Georgia	10,000	500,000
Germany	10,000	1,000,000
Ortì, Italy	10,000	500,000
Serbia	10,000	1,000,000
Spain	10,000	500,000
Mzara Forest, Tunisia	10,000	500,000
Ancestor 1 (NE3a)	10,000	100,000
Ancestor 2 (NE3b)	10,000	100,000
Ancestor 3 (NE1)	10,000	100,000
Divergence/merger time (years)		
tBottleneck	10	200
tRecentIntro	1,000	10,000
t1	1,000	15,000
tTunisia	5,000	20,000
t2	10,000	30,000
t3	20,000	40,000
t4	20,000	50,000
Admixture proportion (from the Spanish Lineage)		
ra	0.001	0.999
Mutation rates (per generation)		
Mean µmic_1	1 × 10^–5^	1 × 10^–4^
Mean pmic_1	0.1	1.0
Mean snimic_1	1 × 10^–8^	1 × 10^–5^

## 4 DISCUSSION

We previously demonstrated that winter moth persisted in at least three glacial refugia (Iberian, Aegean, and Caucasus) during the LGM (Andersen et al., 2017). By analyzing newly sampled populations in North Africa and the Italian Peninsula, we provide evidence for two additional LGM refugia. Contemporary populations sampled in Tunisia and southern Italy differ from all other European populations (Figures 1 and 2) suggesting that the lineages that derived from these refugia did not recolonize Europe following the retreat of glaciers following the LGM (Figure 4). For the southern Italian lineage, it is possible that the Alps presented a significant geographic barrier to their recolonization as has been seen in other systems (see Hewitt, 2000). However, as we do not have samples from central or northern Italy included in this analysis, we can only speculate in regards to barriers to recolonization for this lineage. In regard to the Tunisian lineage, it is very likely that after crossing the Strait of Gibraltar and colonizing northern Africa during the LGM that populations of winter moth became genetically isolated as deciduous forests became geographically isolated in this region with the Mediterranean Sea preventing gene flow with European populations. Again, we can only speculate as to the role of the Mediterranean Sea as a barrier without sampling across northern Africa, southern Spain, and from Mediterranean islands (such as Sicily and Sardinia). However, when our results (i.e., locally distinct populations based on nuclear loci) are combined with mitochondrial studies that have uncovered limited genetic diversity among winter moth populations (Gwiazdowski et al., 2013; Mannai et al., 2017), it suggests that winter moth may be a relatively new addition to the European ecological community, and either became established in Europe prior to the onset of the last glacial cycle, or speciated in Europe shortly before this event.

**Table 4:** Demographic parameter estimates from DiyABC for the best supported scenario presented in Figure 3 representing the relationship of the Mzara Forest, Tunisia population to other sampled localities as presented in Figure 3

Parameters	Mean	Median	Mode	95% CI
Population sizes (effective population size)				
Tbilisi, Georgia	49,800	43,000	39,100	21,300; 121,000
Germany	491,000	468,000	231,000	125,000; 960,000
Ortì, Italy	164,000	133,000	95,300	44,900; 435,000
Serbia	332,000	266,000	225,000	81,100; 866,000
Spain	49,800	41,200	30,000	19,100; 128,000
Mzara Forest, Tunisia	23,300	18,500	15,100	11,000; 70,200
Ancestor 1 (NE3a)	26,700	18,800	11,800	10,500; 82,800
Ancestor 2 (NE3b)	64,000	68,300	84,500	13,700; 98,500
Ancestor 3 (NE1)	32,600	26,400	15,000	10,900; 84,200
Divergence/merger time (years)				
t1	8,900	9,150	9,660	2,480; 14,000
tTunisia	16,200	16,600	18,600	10,100, 19,800
t2	26,600	27,200	27,800	19,900, 29,700
t3	35,000	36,200	38,500	25,200, 39,700
t4	34,200	33,400	31,200	22,900, 48,000
Admixture proportion (from the Spanish Lineage)				
ra	0.746	0.767	0.792	0.397; 0.939
Mutation rates (per generation)				
µmic_1	3.16 × 10^−5^	2.96 × 10^−5^	2.85 × 10^−5^	1.3 × 10^−5^; 6.06 × 10^−5^
pmic_1	0.457	0.433	0.363	0.166, 0.866
snimic_1	2.65 × 10^−7^	9.36 × 10^−8^	1.17 × 10^−8^	1.14 × 10^−8^; 1.41 × 10^−6^

Note

Mean, median, mode, and 95% confidence interval (CI) for each parameter are presented based on the summary of 1 million simulated datasets for the best supported scenario (Scenario 4).

Our results provide evidence that the winter moth population sampled in northwestern Tunisia arrived in North Africa during the LGM. Therefore, factors other than a recent biological invasion are likely responsible for the current outbreak. These factors might include reduced interspecific competition, particularly from other oak defoliators such as *Lymantria dispar* L. (Lepidoptera: Erebidae). *Lymantria dispar* has been reported at outbreak densities in Tunisian oak forests (with four large outbreaks reported between 1925 and 1995; Ben Jemâa, Mnara, Villemant, & Khaldi, 2002); however, no outbreaks of this species have been observed in the region since 2000 (M'nara, 2005). It is therefore possible that the absence of *L. dispar* outbreaks has allowed other defoliators, such as *Tortrix viridana* L. (Lepidoptera: Tortricidae), *Erannis defoliaria* (Clerck) (Lepidoptera: Geometridae)*,* and possibly winter moth to grow to outbreak densities (Mannai, 2017). In addition, climate change in northern Africa might have influenced the population dynamics of winter moth, as already demonstrated in northern Scandinavia (Jepsen, Hagen, Ims, & Yoccoz, 2008). Indeed, northern Tunisia saw dramatic changes in mean annual temperature during the 20th century, with an increase of 2.5–3°C on average, and future climate models predicting an additional increase of 3–4°C coupled with decreased rainfall in the 21st century (Hulme, Doherty, Ngara, New, & Lister, 2001). Environmental factors can play an imortant role in shaping plant–herbivore interactions (Kuglerová, Skuhrovec, & Münzbergová, 2019). It is therefore possible that changes in temperature and/or precipitation might promote winter moth populations either directly by reducing developmental times and/or indirectly by limiting the amount of secondary compounds produced by stressed oak trees. It is also possible that human‐mediated disturbances in the region might be limiting the impact of native natural enemies (such as pathogens, parasitoids, and predators). Further work in this region is needed to understand what factors are resulting in the outbreaks of winter moth in order to preserve these culturally and ecologically important native oak stands.

## 5 CONCLUSIONS

Here, we find the use of two additional glacial refugia, North Africa and southern Italy, by winter moth during the LGM. Our results indicate that populations that utilized both refugia during the LGM diverged from a population that migrated into the Iberian Peninsula, and that subsequently each population became genetically distinct. Curiously, our findings continue to support the results from Andersen et al. (2017) that southern populations appear to be less genetically distinct than northern populations. As such, the biogeographical history of winter moth might represent an important case study for the role of secondary contact in promoting the genetic diversity of outbreaking pest species. Finally, we hope that this new genetic data, and the discovery of additional biogeographic structuring, can be used in conjunction with the recently published winter moth genome (Derks et al., 2015), to promote the use of winter moth as a model system for future research questions, including studies of local adaptation—a subject for which winter moth has previously been studied in the pregenomics era (Buse & Good, 1996; van Dongen, Backeljau, Matthysen, & Dhondt, 1997; Holliday, 1977; Visser & Holleman, 2001).

## CONFLICT OF INTEREST

None declared.

## AUTHOR CONTRIBUTIONS

All authors contributed equally to the study design and manuscript preparation. YM, OE, SD, and MLBJ oversaw sample collection. JCA and NPH conducted analyses. AC and JSE oversaw laboratory work. JSE coordinated the research team.

### OPEN RESEARCH BADGES

This article has earned an https://openscience.com Badge for making publicly available the digitally‐shareable data necessary to reproduce the reported results. The data is available at https://osf.io/yfzh3/?view_only=11a9e5ca6c2743b38abf5aa5fd2bec5f.

## Data Availability

Genotype data for this study is provided as a tab‐delimited file titled “WinterMothTunisiaStructure.txt” uploaded as supplemental material.
