# Supplementary material for: Identification of winter moth (Operophtera brumata) refugia in North Africa and the Italian Peninsula during the last glacial maximum
Source: Ecol Evol. 2019 Nov 18;9(24):13931–41. doi: 10.1002/ece3.5830 (PMC6953680; doi:10.1002/ece3.5830)
Supplement: Supplementary file 1 [file ECE3-9-13931-s001.docx]

**Supplemental Appendix**

Supplemental Table S1. *F*_ST_ values for each population pair (below the diagonal) as well as whether that degree of genic differentiation for each population pair was significantly different than random using the exact *G*-test as implemented in GenePop.

|  | 'Georgia' | 'Germany' | ‘Southern Italy' | 'Serbia' | 'Spain' | 'Tunisia' |
| --- | --- | --- | --- | --- | --- | --- |
| 'Georgia' | 0.0000 | P < 0.0001 | P < 0.0001 | P < 0.0001 | P < 0.0001 | P < 0.0001 |
| 'Germany' | 0.1392 | 0.0000 | P < 0.0001 | P < 0.0001 | P < 0.0001 | P < 0.0001 |
| ‘Southern Italy' | 0.2166 | 0.1090 | 0.0000 | P < 0.0001 | P < 0.0001 | P < 0.0001 |
| 'Serbia' | 0.1425 | 0.0412 | 0.1141 | 0.0000 | P < 0.0001 | P < 0.0001 |
| 'Spain' | 0.2448 | 0.1128 | 0.1972 | 0.1745 | 0.0000 | P < 0.0001 |
| 'Tunisia' | 0.3001 | 0.1973 | 0.2786 | 0.2087 | 0.3023 | 0.0000 |

Supplemental Table S2. Logistic regression results for comparison of DIYABC scenarios that include the divergence of the southern Italy population from other European localities based on different numbers (*n*) of simulated datasets closest to the observed data. Ninety-five percent confidence intervals are presented in brackets.

| *n* | scenario 1 | scenario 2 | scenario 3 |
| --- | --- | --- | --- |
| 3000 | 0.1207 [0.0000,0.6398] | 0.8741 [0.3359,1.0000] | 0.0052 [0.0000,1.0000] |
| 6000 | 0.8924 [0.6815,1.0000] | 0.1046 [0.0000,0.3107] | 0.0031 [0.0000,0.0217] |
| 9000 | 0.9121 [0.7855,1.0000] | 0.0829 [0.0000,0.2037] | 0.0050 [0.0000,0.0116] |
| 12000 | 0.8975 [0.7805,1.0000] | 0.0963 [0.0000,0.2079] | 0.0062 [0.0000,0.0136] |
| 15000 | 0.8778 [0.7616,0.9940] | 0.1149 [0.0038,0.2260] | 0.0073 [0.0000,0.0168] |
| 18000 | 0.8438 [0.7184,0.9692] | 0.1466 [0.0265,0.2668] | 0.0096 [0.0000,0.0241] |
| 21000 | 0.8176 [0.6897,0.9455] | 0.1709 [0.0482,0.2936] | 0.0115 [0.0000,0.0301] |
| 24000 | 0.7986 [0.6720,0.9253] | 0.1881 [0.0666,0.3096] | 0.0133 [0.0000,0.0343] |
| 27000 | 0.7813 [0.6562,0.9065] | 0.2039 [0.0837,0.3240] | 0.0148 [0.0000,0.0382] |
| 30000 | 0.7663 [0.6430,0.8896] | 0.2175 [0.0990,0.3360] | 0.0162 [0.0000,0.0415] |

Supplemental Table S3. Logistic regression results for comparison of DIYABC scenarios that include the divergence of the Tunisian population from other European localities based on different numbers (*n*) of simulated datasets closest to the observed data. Ninety-five percent confidence intervals are presented in brackets.

| *n* | scenario 1 | scenario 2 | scenario 3 | scenario 4 | scenario 5 | scenario 6 | scenario 7 | scenario 8 | scenario 9 |
| --- | --- | --- | --- | --- | --- | --- | --- | --- | --- |
| 9000 | 0.0098 [0.000,1.000] | 0.000 [0.000,1.000] | 0.0465 [0.000,1.000] | 0.9408 [0.7018,1.000] | 0.000 [0.000,1.000] | 0.0025 [0.000,1.000] | 0.000 [0.000,1.000] | 0.0001 [0.000,1.000] | 0.0003 [0.000,1.000] |
| 18000 | 0.0098 [0.000,1.000] | 0.000 [0.000,1.000] | 0.0354 [0.000,1.000] | 0.9502 [0.7983,1.000] | 0.000 [0.000,1.000] | 0.0039 [0.000,1.000] | 0.000 [0.000,1.000] | 0.0001 [0.000,1.000] | 0.0005 [0.000,1.000] |
| 27000 | 0.0081 [0.000,1.000] | 0.000 [0.000,1.000] | 0.0311 [0.000,1.000] | 0.9562 [0.8523,1.000] | 0.000 [0.000,1.000] | 0.0038 [0.000,1.000] | 0.000 [0.000,1.000] | 0.0001 [0.000,1.000] | 0.0007 [0.000,1.000] |
| 36000 | 0.0070 [0.000,1.000] | 0.000 [0.000,1.000] | 0.0277 [0.000,1.000] | 0.9610 [0.8819,1.000] | 0.000 [0.000,1.000] | 0.0035 [0.000,1.000] | 0.000 [0.000,1.000] | 0.0001 [0.000,1.000] | 0.0007 [0.000,1.000] |
| 45000 | 0.0063 [0.000,1.000] | 0.000 [0.000,1.000] | 0.0264 [0.000,1.000] | 0.9631 [0.8970,1.000] | 0.000 [0.000,1.000] | 0.0033 [0.000,1.000] | 0.000 [0.000,1.000] | 0.0001 [0.000,1.000] | 0.0007 [0.000,1.000] |
| 54000 | 0.0058 [0.000,1.000] | 0.000 [0.000,1.000] | 0.0262 [0.000,1.000] | 0.9636 [0.9045,1.000] | 0.000 [0.000,1.000] | 0.0035 [0.000,1.000] | 0.000 [0.000,1.000] | 0.0001 [0.000,1.000] | 0.0007 [0.000,1.000] |
| 63000 | 0.0056 [0.000,1.000] | 0.000 [0.000,1.000] | 0.0268 [0.000,1.000] | 0.9631 [0.9084,1.000] | 0.000 [0.000,1.000] | 0.0037 [0.000,1.000] | 0.000 [0.000,1.000] | 0.0001 [0.000,1.000] | 0.0007 [0.000,1.000] |
| 72000 | 0.0056 [0.000,1.000] | 0.000 [0.000,1.000] | 0.0285 [0.000,1.000] | 0.9611 [0.9075,1.000] | 0.000 [0.000,1.000] | 0.0039 [0.000,1.000] | 0.000 [0.000,1.000] | 0.0001 [0.000,1.000] | 0.0007 [0.000,1.000] |
| 81000 | 0.0057 [0.000,1.000] | 0.000 [0.000,1.000] | 0.0310 [0.000,1.000] | 0.9583 [0.9044,1.000] | 0.000 [0.000,1.000] | 0.0042 [0.000,1.000] | 0.000 [0.000,1.000] | 0.0001 [0.000,1.000] | 0.0007 [0.000,1.000] |
| 90000 | 0.0058 [0.000,1.000] | 0.000 [0.000,1.000] | 0.0331 [0.000,1.000] | 0.9558 [0.9016,1.000] | 0.000 [0.000,1.000] | 0.0044 [0.000,1.000] | 0.000 [0.000,1.000] | 0.0001 [0.000,1.000] | 0.0007 [0.000,1.000] |

Figure S1. DIYABC Scenarios utilized in Step 1 of analyses. 1 = Tbilisi, Republic of Georgia; 2 = Germany; 3 = Ortí, Italy; 4 = Serbia; and 5 = Spain.

Figure S2a. DIYABC Scenarios 1-4 utilized in Step 2 of analyses. 1 = Tbilisi, Republic of Georgia; 2 = Germany; 3 = Ortì, Italy; 4 = Serbia; 5 = Spain; and 6 = Mzara Forest, Tunisia.

Figure S2b. DIYABC Scenarios 5-8 utilized in Step 2 of analyses. 1 = Tbilisi, Republic of Georgia; 2 = Germany; 3 = Ortì, Italy; 4 = Serbia; 5 = Spain; and 6 = Mzara Forest, Tunisia.

Figure S2c. DIYABC Scenario 9 utilized in Step 2 of analyses. 1 = Tbilisi, Republic of Georgia; 2 = Germany; 3 = Ortì, Italy; 4 = Serbia; 5 = Spain; and 6 = Mzara Forest, Tunisia.

Figure S3a. CLUMPAK output for Major Clusters. Population 1 = Tbilisi, Rep. of Georgia; Population 2 = Germany; Population 3 = Ortì, Italy; Population 4 = Serbia; Population 5 = Spain; Population 6 = Mzara Forest, Tunisia.

Figure S3b. CLUMPAK output for Minor Clusters including the divisions of runs to each mode.

Figure S4 – StructureHarvester Results.


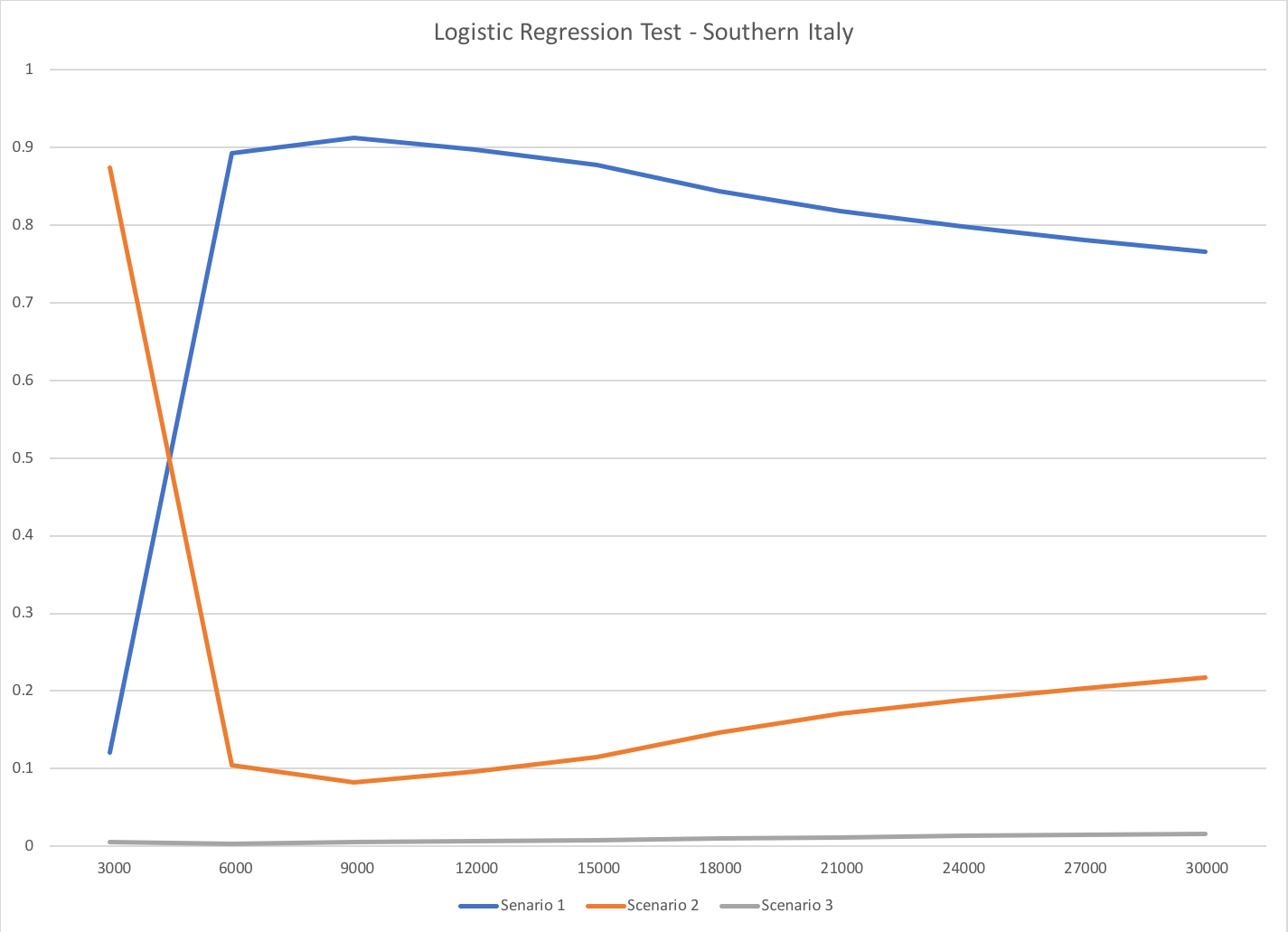


Figure S5. Proportion of simulated datasets (y-axis) for each scenario closest to the real dataset with increasing number of considered simulations (x-axis) using the Logistic Regression Test in DiyABC for scenarios comparing the relationship of the southern Italy population to other sampled European populations.


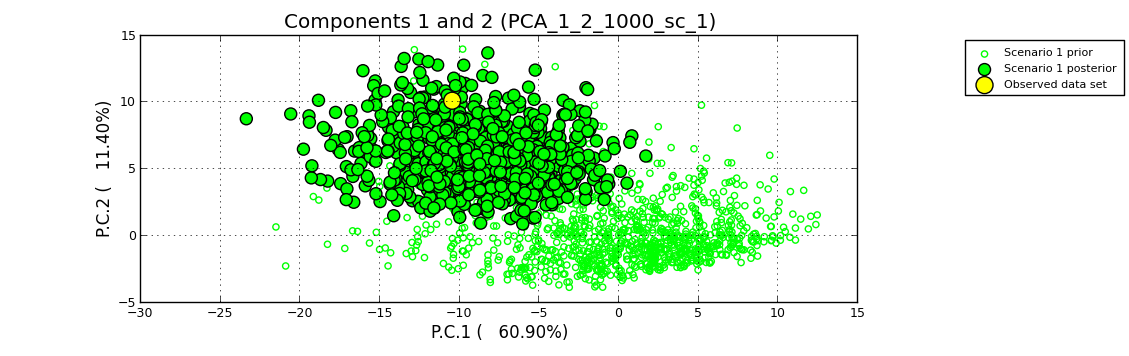


Figure S6. Principle components analysis of the posterior distribution of summary statistics for the best fit scenario comparing the relationship of the southern Italy population to other sampled European populations. The amount of variance explained by principle components 1 and 2 (P.C.1 and P.C.2) and are presented on the x-axis and y-axis, respectively.


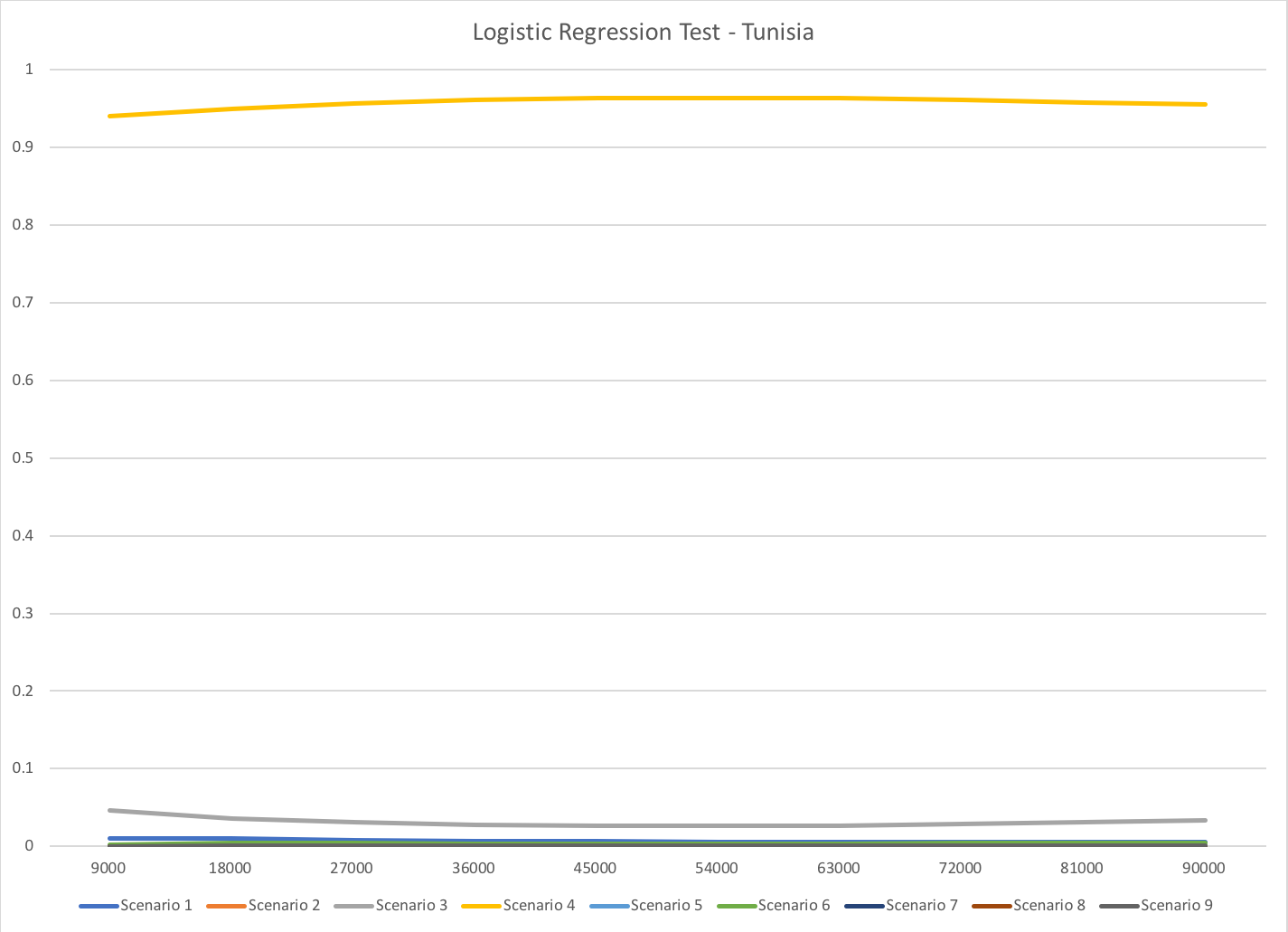


Figure S7. Proportion of simulated datasets (y-axis) for each scenario closest to the real dataset with increasing number of considered simulations (x-axis) using the Logistic Regression Test in DiyABC for the best fit scenario comparing the relationship of the Tunisian population to other sampled European populations.


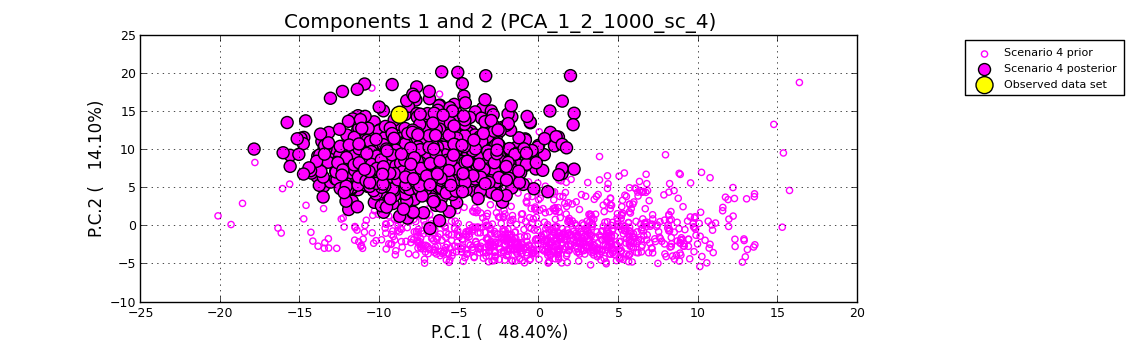


Figure S8. Principle components analysis of the posterior distribution of summary statistics for the best fit scenario comparing the relationship of the Tunisian population to other sampled European populations. The amount of variance explained by principle components 1 and 2 (P.C.1 and P.C.2) and are presented on the x-axis and y-axis, respectively.


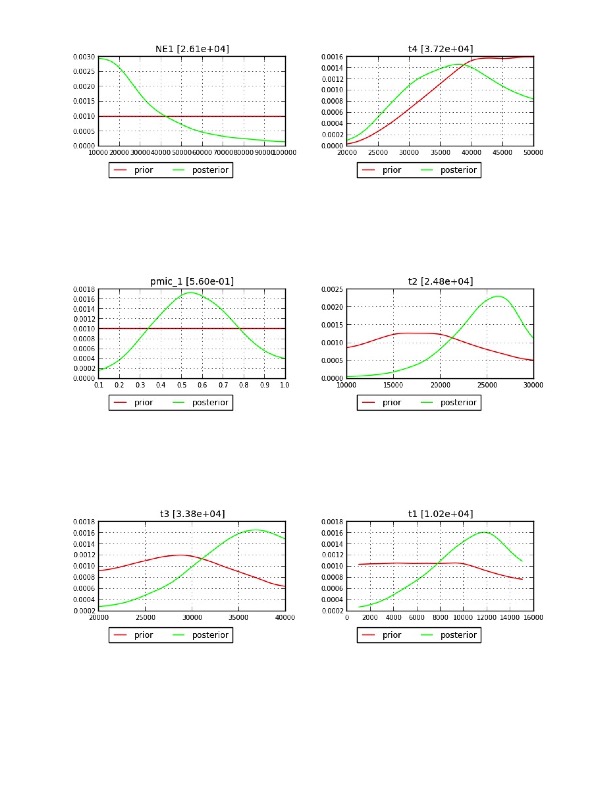
 Figure S9a. Parameter estimates based on the best fit scenario comparing the relationship of the southern Italy population to other sampled European populations.


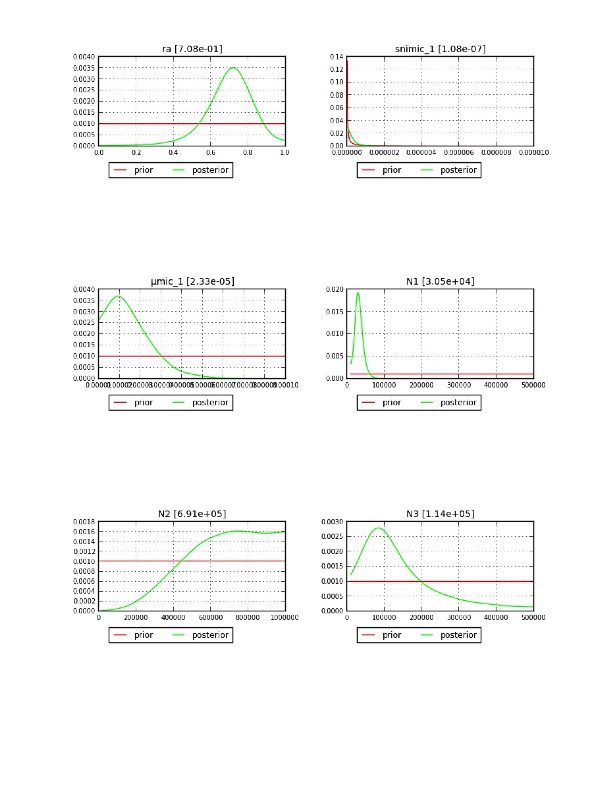
Figure S9b. Parameter estimates based on the best fit scenario comparing the relationship of the southern Italy population to other sampled European populations continued.


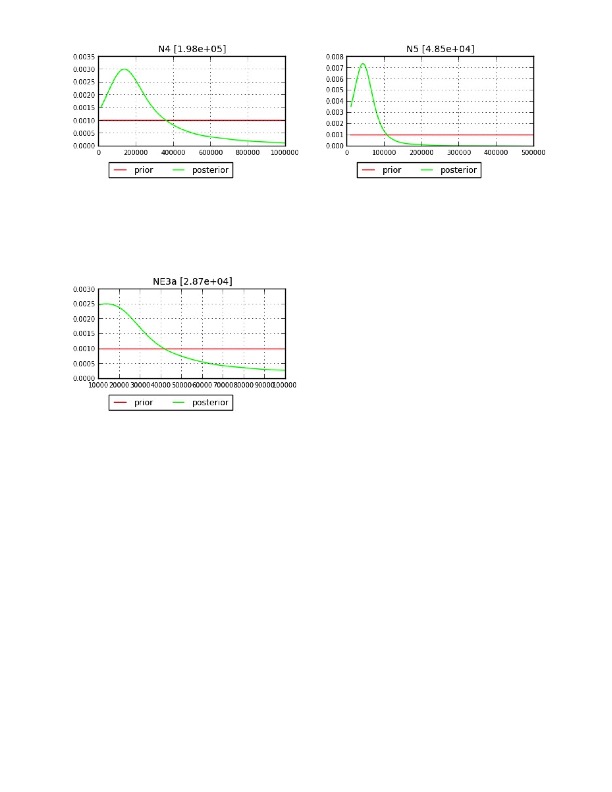
Figure S9c. Parameter estimates based on the best fit scenario comparing the relationship of the southern Italy population to other sampled European populations continued.


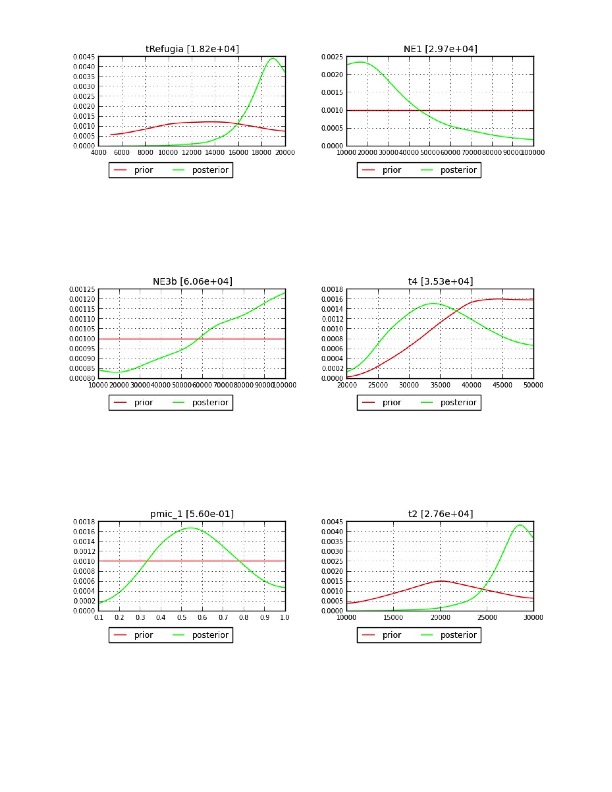
Figure S10a. Parameter estimates based on the best fit scenario comparing the relationship of the Tunisian population to other sampled European populations.


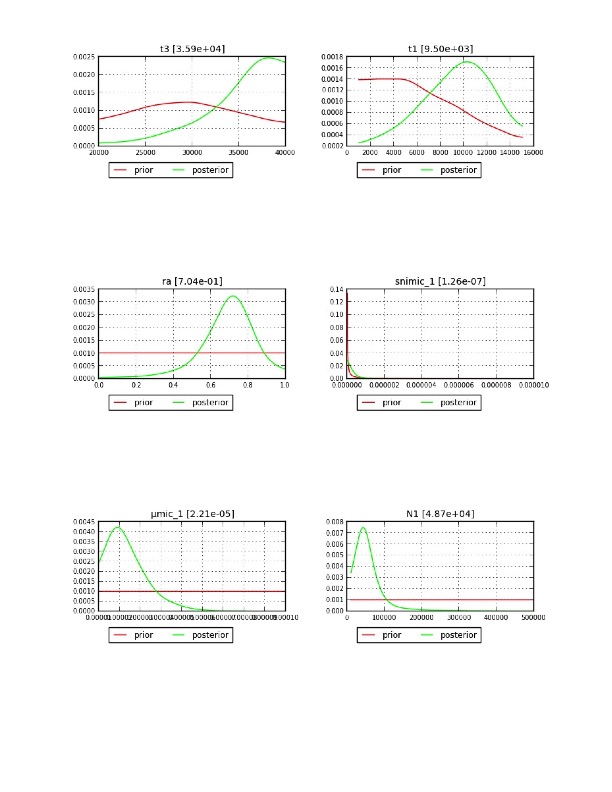
Figure S10b. Parameter estimates based on the best fit scenario comparing the relationship of the Tunisian population to other sampled European populations continued.


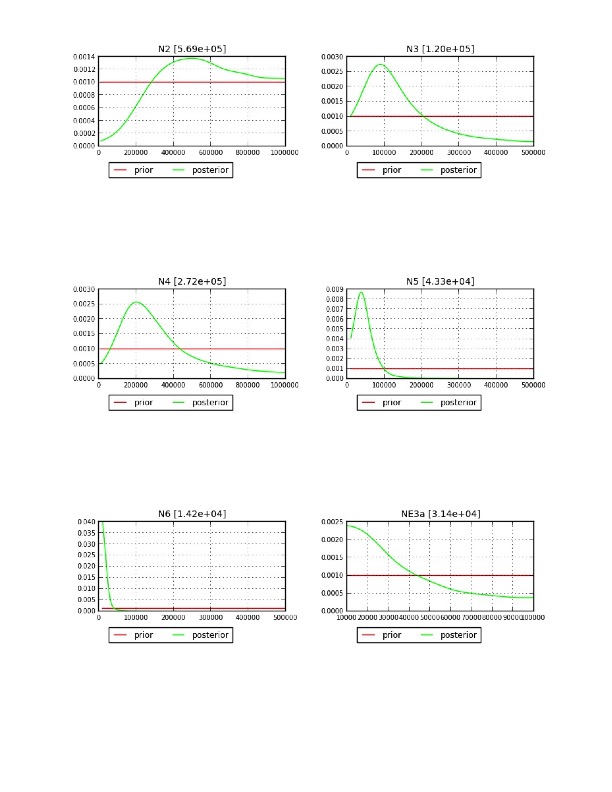
Figure S10c. Parameter estimates based on the best fit scenario comparing the relationship of the Tunisian population to other sampled European populations continued.
